# Efficient pH and dissolved CO_2_ conditions for indoor and outdoor cultures of green alga *Parachlorella*

**DOI:** 10.3389/fbioe.2023.1233944

**Published:** 2023-09-11

**Authors:** Akari Takagi, Misato Nagao, Yuya Uejima, Daisaku Sasaki, Munehiko Asayama

**Affiliations:** ^1^ College of Agriculture, Ibaraki University, Ibaraki, Japan; ^2^ BioX Chemical Industries Co., Ltd., Hiroshima, Japan; ^3^ United Graduate School of Agricultural Science, Tokyo University of Agriculture and Technology, Tokyo, Japan

**Keywords:** algae, biorefinery, carbohydrate, lipid, *Parachlorella*, polysaccharide

## Abstract

Efficient pH and dissolved CO_2_ conditions for indoor (50–450 mL scale) and outdoor (100–500 L scale) culture of a green alga BX1.5 strain that can produce useful intracellular lipids and extracellular polysaccharides were investigated for the first time in *Parachlorella* sp. The cultures harvested under 26 different conditions were analysed for pH, dissolved CO_2_ concentration, and the biomass of extracellular polysaccharides. The BX1.5 strain could thrive in a wide range of initial medium pH ranging from 3 to 11 and produced valuable lipids such as C16:0, C18:2, and C18:3 under indoor and outdoor culture conditions when supplied with 2.0% dissolved CO_2_. Particularly, the acidic BG11 medium effectively increased the biomass of extracellular polysaccharides during short-term outdoor cultivation. The BG11 liquid medium also led to extracellular polysaccharide production, independent of acidity and alkalinity, proportional to the increase in total sugars derived from cells supplied with high CO_2_ concentrations. These results suggest *Parachlorella* as a promising strain for indoor and outdoor cultivation to produce valuable materials.

## 1 Introduction

Microalgae have attracted attention as producers of valuable materials, such as lipids, carbohydrates (sugars), proteins, and pigments, because of their high carbon dioxide (CO_2_) fixation capacity (Kumar et al., 2020; Okeke et al., 2022; Olguín et al., 2022). Besides, low-cost indoor and outdoor culture conditions are essential for large-scale biorefineries using microalgae (Franz et al., 2013; Laurens et al., 2017; Paliwal and Jutur, 2021). In addition to the culture medium composition (nitrogen, phosphorus, and potassium), factors such as the pH of the medium (Doney et al., 2009; Lam and Lee, 2012; Sakarika and Kornaros, 2016; Khan et al., 2018), dissolved CO_2_ concentration (Santos et al., 2012), and light type (sunlight or artificial light) and intensity vary greatly depending on the strain used for microalgal cultivation under general autotrophic culture conditions. Furthermore, the specifications of the culture equipment substantially impact biomass yield. Thus, effective biorefineries are not viable unless the algal species used, bioreactor specifications (open pound, pipe reactor, tube, flat panel, solid phase, etc.), media composition, and inducing culture conditions related to producing valuable materials are well-matched.

The novel green alga *Parachlorella* sp. BX1.5 strain was isolated from an outdoor culture comprising natural water collected in Hiroshima, Japan (Sasaki et al., 2020). The BX1.5 strain, which was cultured using the BG11 medium (Rippka, 1988) and was extensively characterised, can significantly accumulate intracellular lipids, such as C16:0, C18:2 (ω6), and C18:3 (ω3) with potential use as biodiesel and edible oil, and simultaneously produce extracellular polysaccharides that are novel ultra-high molecular weight (1.75 × 10^6^ Da) linear rhamnans, providing excellent viscosity, emulsion stability, and stress resistance (Sasaki et al., 2020; Sasaki et al., 2021). Green algae produce abundant sugars (carbohydrates) and lipids as triglycerides under specific conditions (Hu et al., 2008; Msanne et al., 2012; Khan et al., 2018; Sasaki et al., 2020). Efficient cultivation systems are promising for the social implementation of biorefineries. Therefore, from the cost standpoint, it is vital to grow microalgae efficiently in a short period while suppressing the growth of contaminants under acidic or alkaline pH medium conditions and producing useful substances inside and outside cells in large-scale outdoor culture systems using sunlight. The pH of the culture medium for microorganisms in fermenters also affects the dissolution rate of CO_2_ gas in the medium, and the efficiency of CO_2_ supply to the liquid phase increases on the strongly alkaline side (Santos et al., 2012; Sakarika and Kornaros, 2016; Sakarika and Kornaros, 2017). However, little is known about the effects of pH and dissolved CO_2_ concentration of the medium on the growth and productivity of the green alga *Parachlorella* sp. in outdoor culture.

Therefore, in this study, we evaluated 18 combinations of pH and dissolved CO_2_ concentration for indoor (bench scale) cultivation using the novel green alga *Parachlorella* sp. strain BX1.5. Based on the indoor results, eight additional culture conditions were selected to identify conditions for the efficient and rapid culture of the green alga *Parachlorella* sp., even when scaled up outdoors (large-scale production).

## 2 Materials and methods

### 2.1 The strain and cultivation conditions

**FIGURE 1:**
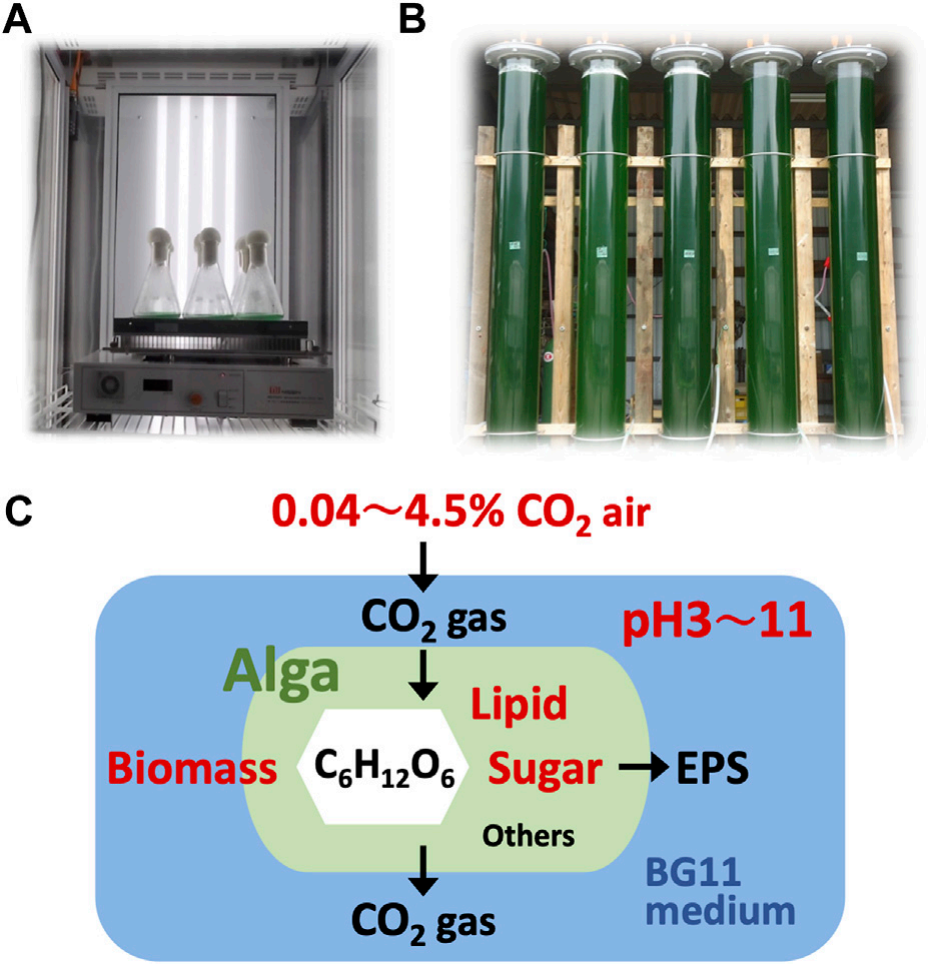
Cultivation systems. Green algae *Parachlorella* sp. cells were cultivated indoors **(A)** and outdoors **(B)**; CO_2_ gas was supplied at 0.04, 2.0, or 4.5% into BG11 medium initially adjusted to pH 4 (or 3), 8, or 11. Dissolved CO_2_ gas in the culture and products such as biomass, lipids, and sugars were measured and evaluated **(C)**.

The green alga *Parachlorella* sp. strain BX1.5, whose 18S rDNA has been published on DDBJ (LC473527), has been isolated, and the production of valuable lipids and extracellular polysaccharides has been reported (Sasaki et al., 2020; Sasaki et al., 2021). The cells were pre-cultivated indoors in 50 mL BG11 medium (Rippka, 1988) in 100-mL Erlenmeyer flasks for 5 days. The flasks were placed in a cultivation chamber CF-415 (TOMY Company Ltd., Tokyo, Japan), supplied with 0.04, 2.0, and 4.5% CO_2_, and subjected to reciprocating-shaking at 40 rpm under continuous white-light exposure at 100 μmol photons m^-2^ s^-1^ (Figure 1A). Approximately 5 mL of the cell culture was collected, and the cells were suspended in 50 mL of new BG11 media in 300-mL Erlenmeyer flasks. The BG11 medium (pH 8) was adjusted to pH 4 or 11 with hydrochloric acid (HCl) or sodium hydroxide (NaOH), respectively. The flasks were placed in the same cultivation chamber used for the main culture. After 1, 2, or 3 days, the cells were collected.

For outdoor cultivation, 50 mL of the BX1.5 strain culture medium was added to 5 L autoclaved BG11 liquid medium in a 10 L glass tank as a pre-culture. The tank was then aerated with 2.0% CO_2_ gas and stirred under natural light for 10 days. The BG11 liquid medium for this culture was prepared by pouring all BG11 reagents into a tank filled with 500 L (five 100 L medium tanks) of tap water and adjusting the pH to 3 or 11 by adding HCl or NaOH, respectively. A vertical pipe reactor (VPR, Φ25 cm × 210 cm) served as the main incubation; 100 L of the BG11 liquid medium after pH preparation was fed through a 1 µm filter. In the case of the BX1.5 strain, 1 L of the pre-culture medium was added to the VPR at the start of the main cultivation, and the culture was continued at approximate 700 to 1,800 μmol photons m^−2^ s^−1^ of sunlight for 14 days under the supplementation of 0.04 or 2.0% CO_2_ gas at 60 L/min (Figure 1B). One litre of culture medium was collected from the lower valve of the VPR on days 0, 7, and 14 and these samples were stored at −60°C until used for material production measurement.

### 2.2 Measuring culture pH, dissolved carbon dioxide concentration, and biomass

**FIGURE 2:**
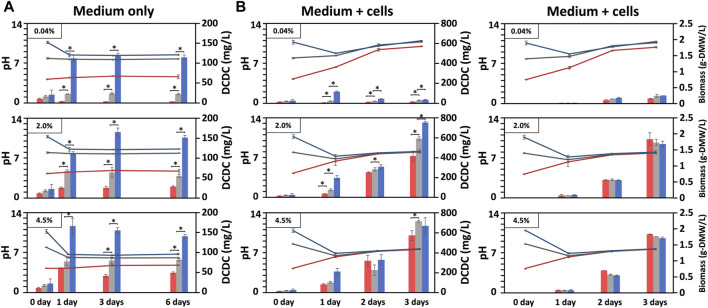
Biomass produced under indoor conditions with varying pH and DCDC. Changes in BG11-culture pH and dissolved CO₂ concentration (DCDC, the sum of HCO₃⁻ and CO₃^2^⁻ ions) in the media supplied with CO₂ indoors, in which BG11 medium without **(A)** or with BX1.5 cells **(B)** cultivated for 3 or 6 days under pH conditions adjusted to 4, 8, or 11 in an incubation chamber supplied with 0.04, 2.0, or 4.5% CO₂. The pH (line graph: red, pH 4; grey, pH 8; blue pH 11), DCDC (ppm as mg/L-culture for bar graph: red, pH 4; grey, pH 8; blue pH 11), and biomass (g-DMW as grams of dried biomass weight for bar graph: red, pH 4; grey, pH 8; blue pH 11) were sequentially measured. Error bars indicate three independent experiments. *P, Student’s *t*-test: *p < 0.05.

**FIGURE 3:**
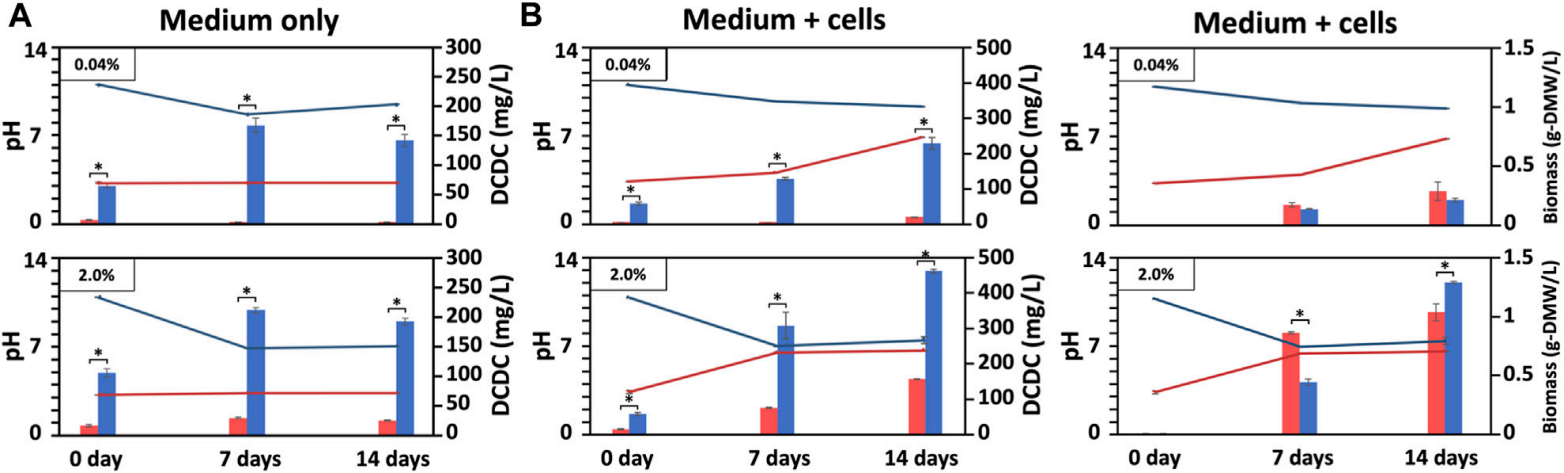
Biomass produced under outdoor conditions with varying pH and DCDC. Changes of BG11-culture pH and dissolved CO₂ concentration (DCDC, the sum of HCO₃⁻ and CO₃^2^⁻ ions) in the media supplied with CO₂ outdoors, in which BG11 medium without **(A)** or with BX1.5 cells **(B)** cultivated for 14 days under outdoor pH conditions adjusted to 3 or 11 supplied with 0.04 or 2.0 of CO₂ in Hiroshima prefecture in Japan (August in 2020). The pH (line graph: red, pH 3; blue pH 11), DCDC (ppm as mg/L-culture for bar graph: red, pH 3; blue pH 11), and biomass (g-DMW as grams of dried biomass weight for bar graph: red, pH 3; blue pH 11) were sequentially measured. Error bars indicate three independent experiments. *P, Student’s *t*-test: *p < 0.05.

The culture pH, dissolved carbon dioxide concentration (DCDC), and biomass (Supplementary Table S1, Figures 2, 3) were measured. To measure the pH, 50 mL of the supernatant of the BG11 medium or BX1.5 culture was harvested, and the pH was measured (LAQUA F-71: Horiba Co., Ltd., Kyoto, Japan). To measure DCDC, 50 mL of the BG11 medium or BX1.5 culture supernatant was harvested and measured using a portable carbon dioxide gas concentration metre (Model CGP-31, DKK-TOA Co., Ltd., Tokyo, Japan). The 50 mL of BX1.5 culture were harvested, centrifuged (8,000 × *g*, 10 min, 10°C), and the cell biomass containing the BX1.5 extracellular polysaccharide (bxEPS) was stored at −80°C until use. The cell matter was freeze-dried as DCM (dried cell matter with bxEPS) in a vacuum freeze dryer (FZ-Compact; Labconco Co., Ltd., Kansas City, United States of America) for 72 h. The weight of DCM (DMW) was measured using an electron precision balance (MS603S; METTLER TOLEDO, Tokyo, Japan). Of note, since the supernatant and cells cannot be separated by centrifugation due to the abundant bxEPS production, 20 mL of the cell culture was directly transferred to a 50 mL tube, frozen at −80°C, and lyophilised as the DCM sample.

### 2.3 Cell staining and microscopic observation

**FIGURE 4:**
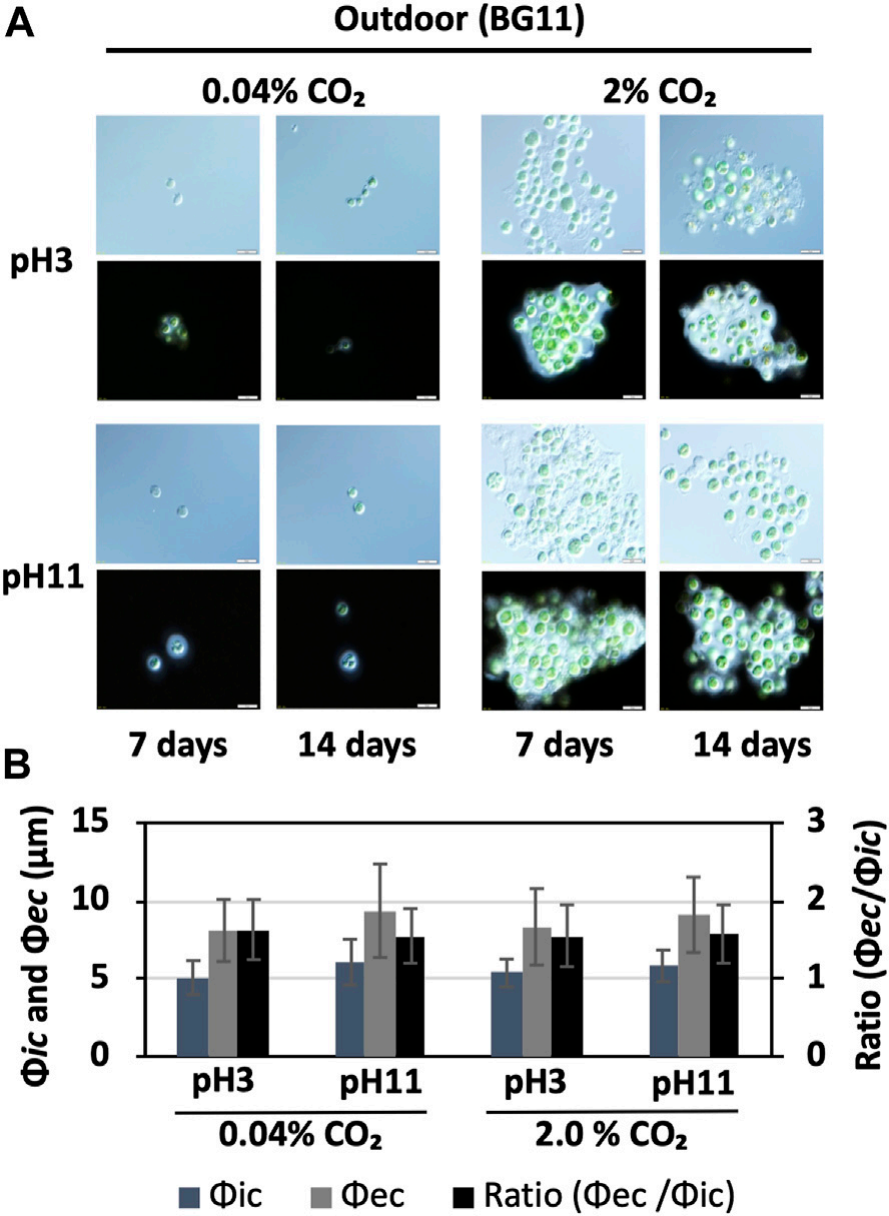
Cell size and EPS production under outdoor conditions. **(A)** BX1.5 cells cultivated under the specified condition for 14 days, as shown in Figure 3, were harvested, and an aliquot of the culture was observed under a microscope without (top) or with (bottom) India ink. **(B)** Diameters (μm) of the cells (Φ*ic*) and cell + EPS (Φ*ec*) are indicated on the left of panel A (bottom, 14 days), and the ratios (Φ*ec*/Φ*ic*) are shown on the right. Sample conditions: CO₂ 0.04%, pH 3, *n* = 21; CO₂ 0.04%, pH 11, *n* = 20; CO₂ 2.0%, pH 3, *n* = 23; CO₂ 2.0%, pH 11, *n* = 23, respectively.

To detect bxEPS, a 100-μL aliquot of cell culture, harvested from the main culture, was suspended in 20 μL of India ink (Daiso Co., Ltd., Japan) and observed under an optical microscope (BX53; Olympus, Tokyo, Japan) with differential interference contrast/half of light and a shutter speed of 0.48 s (Sasaki et al., 2020; Sasaki et al., 2021). Based on this observation, the diameters inside (Φ*ic*) and outside (Φ*ec*) of the cells with bxEPSs were measured (Figure 4). To check for cell contamination in the outdoor culture, a portion of the cell culture was collected and microscopically observed, ensuring that bacterial contamination was less than 0.1% in a single field of view at ×100 and ×1,000 magnification.

### 2.4 Analysis of BX1.5 extract sugar production

**FIGURE 5:**
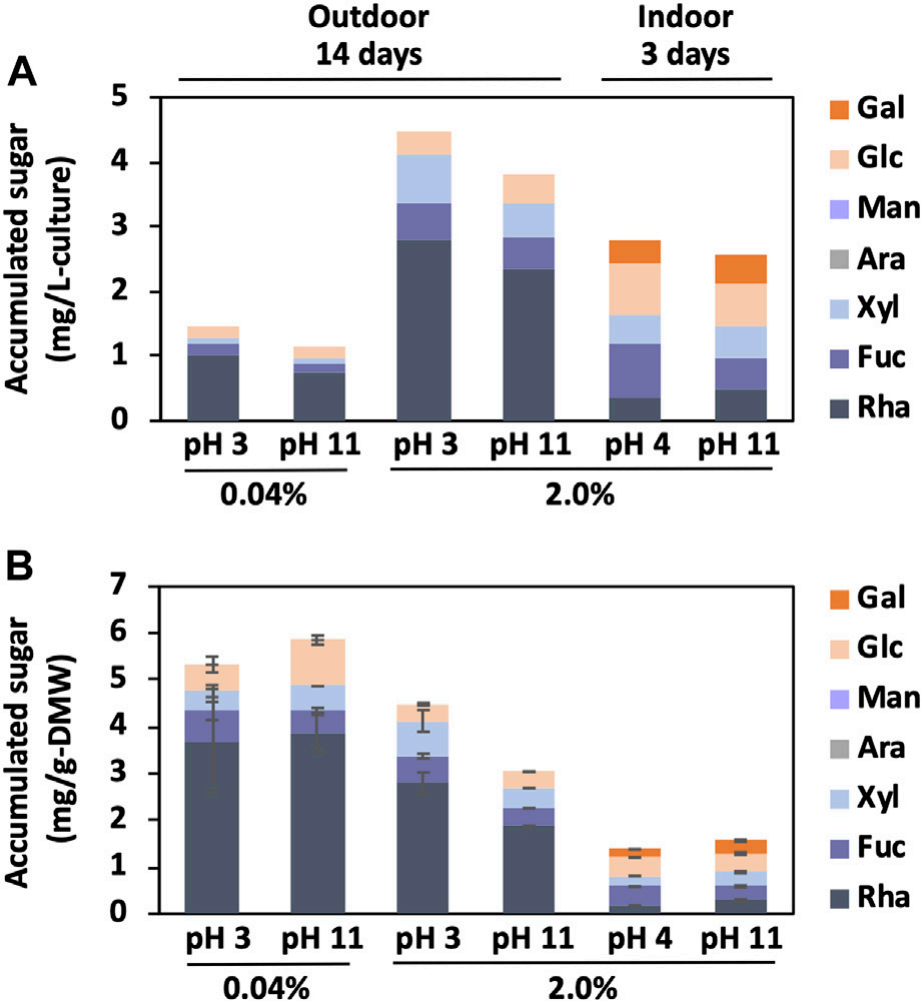
Sugar amounts from BX1.5 cells. The neutral monosaccharide content derived from the cultivation conditions (Figures 2, 3) was measured using the phenol-sulfuric acid method. Values are shown as FAMEs per 1 L of medium volume **(A)** or 1 g of DMW **(B)**, respectively. Values are presented as mean ± standard deviation of three independent experiments **(B)**.

Since the BX1.5 extract with bxEPS contains galactose, glucose, rhamnose, and xylose (Sasaki et al., 2021), the main carbohydrate (sugar) content of the BX1.5 extract was measured using the phenol-sulfuric acid method (Dubois et al., 1956), using rhamnose as a reference. The ground samples (0.05 g) were dissolved in 1 mL of 72% (v/v) sulfuric acid and allowed to stand for 2 h at 20°C to measure the neutral sugar composition of BX1.5 extracts. The resultants were added to 8 mL of water and hydrolysed at 105°C for 20 h to obtain neutral monosaccharides. After adding barium carbonate to neutralise the hydrolysate, the collected aqueous phase was centrifuged at 10,000 × *g* for 10 min, leading to precipitation. The supernatant was filtered through a USY-1 filter (Advantec Co., Ltd., Tokyo, Japan) and freeze-dried. The neutral sugar content was measured using high-performance liquid chromatography (HPLC) on a Shimadzu 10Avp chromatography system (Shimadzu Co., Ltd., Kyoto, Japan) equipped with an NH2P-50 4E column (Φ4.6 mm × 250 mm). The eluent was 80% acetonitrile (CH_3_CN) with 250 mM phosphate at a 0.8 mL/min flow rate. Thirty microlitres of the sample, dissolved in ultrapure water and filtered through a cellulose acetate filter (0.45 µm; Advantec Co., Ltd.), was injected into the column. Monosaccharide standards, such as arabinose, fucose, galactose, glucose, rhamnose, and xylose, were obtained from Nacalai Tesque, Inc. (Kyoto, Japan). The total sugar content was calculated based on relative monosaccharide content (mol%, w/w) referring to total monosaccharides of 100% (Figure 5).

### 2.5 Thin-layer chromatography analysis of lipids

**FIGURE 6:**
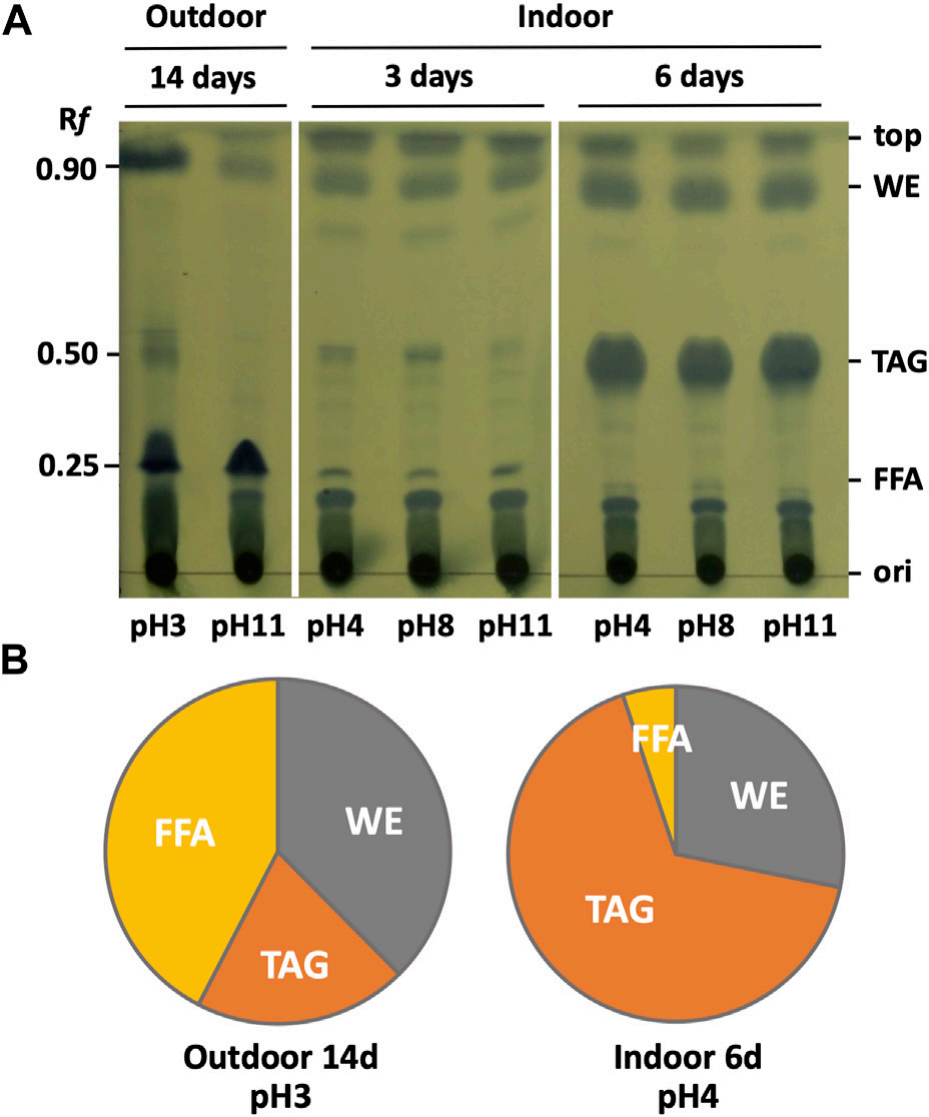
TLC analysis of intracellular lipids. **(A)** Total lipids were extracted from BX1.5, grown in Figures 2, 3, and subjected to TLC analysis. R*f* values and relative mobility. WE, wax ester; TAG, triacylglycerol; FFA, free fatty acid. **(B)** Band intensities corresponding to lipids on the TLC plate were quantified using an imaging analyser and expressed as a percentage of the amount of WE, TAG, and FFA, respectively.

Samples for thin-layer chromatography (TLC) were prepared as described previously (Sasaki et al., 2020) with slight modifications. BX1.5 cells in 50 mL of culture medium grown under the conditions shown in Figures 2, 3 were freeze-dried, and the dried matter was transferred to 15 mL volumetric glass tubes to which 3 mL of extraction solvent (chloroform: methanol = 2:1) was added. After sonication using an ultrasonic disruptor (UD-10 at output level 70 for 10 min; TOMY SEIKO Co., Ltd., Tokyo, Japan), the solution was transferred to a 50 mL tube and left at −30°C for 3 h. After centrifuging the extract at 10°C and 8,000 × *g* for 10 min, the upper layer was collected, and an equal volume of 0.9% KCl solution was added and stirred. After centrifugation (8,000 × *g* at 15 °C for 10 min), the lower layer (organic layer) was collected using a Pasteur pipette and transferred to a 30 mL glass test tube. The organic layer was concentrated and dried using a rotary evaporator (V-850; BUCHI, Flawil, Switzerland), and the dried material was dissolved in 200 µL chloroform to prepare a sample for TLC. Of the 20 µL of the prepared sample, 4 µL was spotted onto a silica gel plate (#60F254; Merck Millipore, Darmstadt, Germany), placed in a development tank, and developed with a development solution (hexane: diethyl ether: acetic acid = 80:20:1). After expansion, the solvent front was marked with a pencil, and the TLC plate was dried entirely. The dried TLC plate was soaked in 5% (w/v) phosphomolybdate in ethanol for a few seconds and then dried by heating with a hair dryer (Figure 6A) to stain the lipids. Band intensities corresponding to lipids on the TLC plate were quantified using an imaging analyser (BIO-1D system; Vilber Lourmat Co., Ltd., Cedex, France) and expressed as a percentage of the amount of triacylglycerol (TAG), free fatty acid (FFA), and wax ester (WE; Figure 6B).

### 2.6 Gas chromatography with flame-ionisation detection analysis of lipids

Samples for gas chromatography with flame-ionisation detection (GC-FID) were prepared as previously described (Sasaki et al., 2020) with slight modifications. Samples were incubated and collected under the conditions shown in Figures 2, 3 and dried in a vacuum freeze dryer for 72 h. The dried matter was transferred to a 30 mL glass tube, 4 mL of extraction solvent (chloroform: methanol = 2:1) was added, and 10 µL of eicosanoic acid [CH_3_(CH_2_)_18_COOH, molecular weight 312.54] solution prepared at 12,000 × *g* was added as an internal standard (the final sample was dissolved in 1 mL of hexane, so the final concentration was 100 ppm) Bacteria with the extraction solvent and eicosanoic acid were sonicated using an ultrasonic disruptor (UD-10) at an output level of 70 for 10 min. The samples were then centrifuged in a large centrifuge at 6,500 × *g* at 10°C for 10 min, and the supernatant liquid was transferred to a new 50 mL tube. An equal volume (4 mL) of 5% (w/v) sodium chloride solution was added to the supernatant solution, shaken vigorously up and down 100 times, centrifuged at 7,000 × *g* at 10°C for 10 min, and the lower layer (organic layer) was transferred into a 30 mL glass sliding test tube using a Pasteur pipette. The organic layer was concentrated and dried in a rotary evaporator. The dried product was dissolved in 1 mL of toluene and transferred to a glass tube with a lid. Then 2 mL of HCl (5%–10%) methanol solution (Tokyo Chemical Industry Co., Ltd., Tokyo, Japan) was added to the extract, stirred, and reacted in a water bath at 80–90°C for 3 h to convert fatty acids derived from fats and oils into methyl esters (fatty-methyl esters; FAMEs). These products were transferred to 50 mL tubes. The lidded glass tube was washed with 1 mL of sterile distilled water and 2 mL of hexane. The washing solution was combined with the extraction solution in a 50 mL tube, and the aqueous and organic layers were thoroughly mixed by shaking vigorously up and down 100 times. After centrifugation at 7,000 × *g* at 15°C for 10 min, the upper layer (organic layer) was transferred to a 30 mL glass test tube using a Pasteur pipette, concentrated in an evaporator to less than 1 mL, transferred to a 1.5 mL vial for GC-FID, and combined with hexane in a washed glass test tube to prepare the final volume to 1 mL.

To determine the retention times of methyl-esterified C16:0–16:3, C18:0−C18:3, and C20:0 (eicosane), samples of each fatty acid and eicosane purchased from Sigma-Aldrich (St. Louis, MO, United States of America) were analysed on a GC-2014 system from Shimadzu Co., Ltd., Kyoto, Japan. For methyl-esterified samples, 20–100 µL were mixed with hexane, and the mixture was mixed up to 1 mL with hexane and used as the sample for FID. For samples of fatty-methyl esters (FAMEs), 10 µL was mixed with 1 mL of toluene and 2 mL of HCl (5%–10%) methanol solution (Tokyo Chemical Industry Co., Ltd., Tokyo, Japan) to prepare a sample for FID.

**FIGURE 7:**
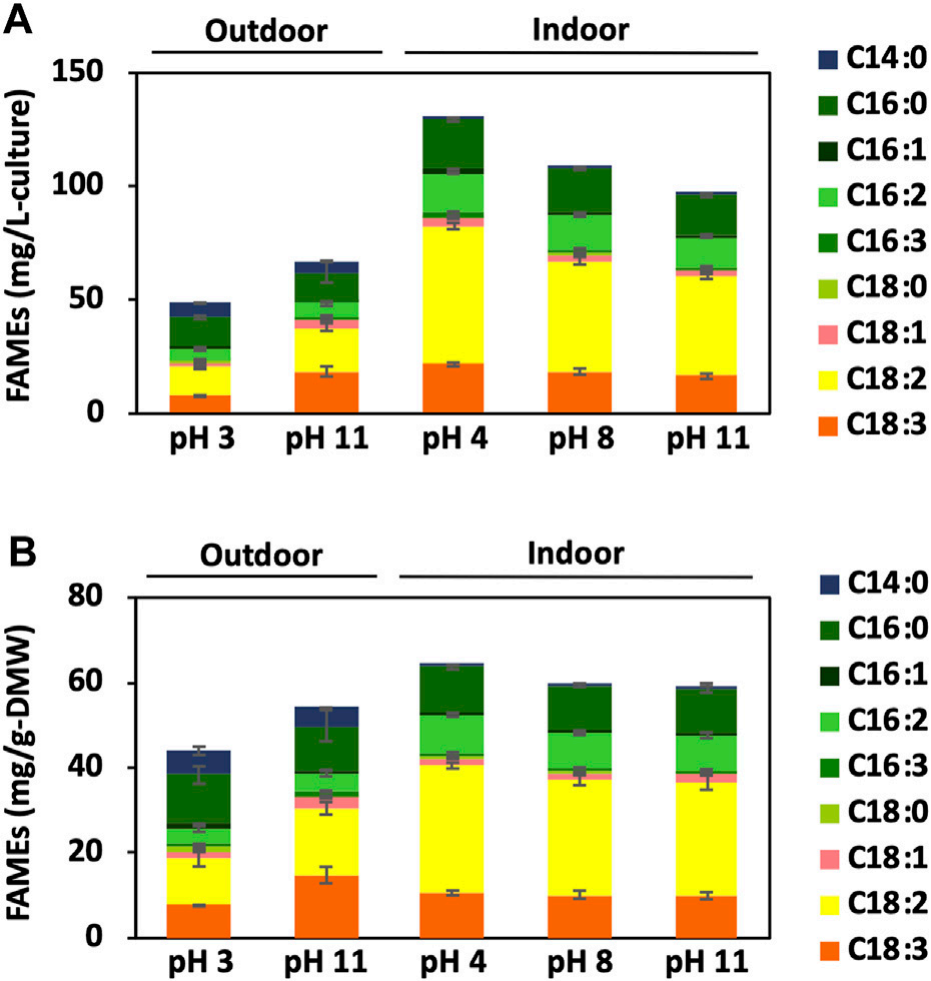
FAME accumulation in BX1.5 cells. Fatty-acid methyl esters (FAMEs) produced in the cells cultivated under indoor (3 days) or outdoor (14 days) conditions supplied with 2.0% CO₂-air were analysed using GC-FID. Values are shown as FAMEs per 1 L of medium volume **(A)** or 1 g of DMW **(B)**, respectively. Values are presented as mean ± standard deviation of three (indoor) or two (outdoor) independent experiments.

The samples for FID were analysed using a Shimadzu GC-2014 system, and the ratio to eicosanoic acid was calculated from the peak area at each retention time. Furthermore, the FAME concentration in the sample was determined from the ratio to the internal standard eicosane (final concentration of 100 ppm), and the productivities were expressed per litre of culture medium (mg/L-culture) and Gram weight of dried cell matter (mg/g-DMW; Figure 7).

### 2.7 Statistical analysis

Statistical analysis was performed using a Student’s t-test (Saito et al., 2022), which was used to calculate the *p*-value and compare differences between samples. Differences were considered statistically significant at *p* < 0.05.

## 3 Results and discussion

### 3.1 Indoor cultivation conditions

The objective of this study was to find suitable conditions for indoor and outdoor large-scale culture and to provide a reference for microalgal biorefineries in light of the growth potential of *Parachlorella* sp. BX1.5 and its ability to produce valuable lipids and extracellular polysaccharides (Sasaki et al., 2020; Sasaki et al., 2021). We first optimised the indoor culture conditions and then selected the most promising results to test suitable outdoor culture conditions. Therefore, we set up 18 different indoor culture conditions with different medium pH (4, 8, and 11), CO_2_ supply concentrations (0.04%, 2.0%, and 4.5%), and with or without cell addition (Table 1). We then evaluated the pH, DCDC in the medium, and biomass production over time (Figure 1A). First, the medium fixation rate of supplied CO_2_ was examined by changing the initial medium pH and CO_2_ concentration conditions under “cell-free” indoor culture (Figure 2A). When the shaking culture was started, the pH of the alkaline medium decreased from 11 to near-neutral conditions (pH 8) on day 1 under all CO_2_ conditions and was maintained at pH 8 until day 6. This result is because carbon dioxide dissolves in the medium as bicarbonate (HCO_3_^−^) or carbonate ions (CO_3_^2-^), thereby lowering the pH of the medium (Doney et al., 2009). DCDC levels were highest at pH 11 for all CO_2_ concentration conditions. Even in the 4.5% CO_2_ condition, CO_2_ levels were 2.7 times higher than at pH 4 and 1.7 times higher than at pH 7 (pH 4, 47.33; pH 8, 79.33; pH 11, 136.66 mg/L-culture/6 days). This supports a previous report showing dissolved CO_2_ gas levels increase in alkaline liquid media (Santos et al., 2012). These results showed that the amount of dissolved CO_2_ gas depends on the medium pH and the CO_2_ concentration supplied. Furthermore, a comparison of DCDC results obtained after supplying 2% and 4.5% CO_2_ gas suggests that HCO_3_^−^ and CO_3_^2-^ in the culture medium were almost saturated under the 2.0% CO_2_ gas supply conditions at pH 8 and 11 but increased at pH 4 under the 4.5% CO_2_ gas supply conditions.

**TABLE 1:** Biomass production of *Parachlorella* strain BX1.5 under various incubation conditions of pH and CO_2_ gas supplying.

Location	Incubation scale	Combination conditions	CO_2_ gas (%)^a^	Culture initial pH	Culture type	Dissolved CO_2_ in culture (mg/L)^b^	Biomass production (g-DMW/L/day)^c^
Indoor	50 mL^d^	9	0.04	4	BG11 only	∼150@pH11	-
			2.0	8			
			4.5	11			
		9	0.04	4	BG11+ cells	∼747@pH11	0.61@pH4(1.83 g/L/3 days)
			2.0	8			
			4.5	11			
Outdoor	100 L^e^	4	0.04	3	BG11 only	∼150@pH11	-
			2.0	11			
		4	0.04	3	BG11+ cells	∼447@pH11	0.12@pH3(0.83 g/L/7 days)
			2.0	11			

^a^ Indoor, CO_2_ gas is constantly filled in an incubator; Outdoor, CO_2_ gas is constantly blown into an incubator.

^b^ The highest value of DCDC under the nine incubation conditions.

^c^ The highest value of biomass (cells with EPS) production under the four incubation conditions.

^d^ Amount of media per an Erlenmeyer flask.

^e^ Amount of media per a vertical pipe reactor (VPR).

Next, the BX1.5 strain was added to the BG11 liquid medium and cultured with “BX1.5 cells” under shaking while supplying CO_2_ at various concentrations in the same manner. pH and DCDC in the culture medium were also measured (Figure 2B). Notably, the convergence of the medium pH was more pronounced, with a pH of 10 at 0.04%, pH 8 at 2.0%, and pH 7.5 at 4.5%. The difference in the amount of DCDC for each initial pH condition was small compared with the condition in which only the culture medium was shaken (Figure 2A). In the pH 11 medium with 0.04% CO_2_, the pH of the medium decreased to near-neutral and then gradually became alkaline, possibly because the CO_2_ gas consumption in the medium during photosynthesis exceeded the rate of CO_2_ gas dissolution into the medium. The amount of DCDC was approximately 1/4 times lower than in the 30.7 mg/L-culture medium and the day 3 conditions without cell addition. In contrast, under 2.0% and 4.5% CO_2_ conditions, the amount of dissolved CO_2_ gas was 4.5 times higher in both pH media than under the no cell addition condition (2.0%: pH 4, 410; pH 8, 586.6; pH 11, 746.7 mg/L-culture/3 days vs. 4.5%: pH 4, 573; pH 8, 716.7; pH 11, 670 mg/L-culture/3 days). This result was presumably due to the increased respiration of the BX1.5 strain as BX1.5 cells proliferated. The CO_2_ gas derived from respiration was also dissolved into the medium (Figure 1C). In the same way as in the condition with no cells, there was almost no difference in the amount of DCDC between the 2.0% and 4.5% CO_2_ conditions when the BX1.5 strain was cultured. This result indicates that the amount of DCDC obtained under the 2.0% CO_2_ conditions did not change when the supplied CO_2_ concentration was increased to 4.5% or above (Figure 2B, left).

The result in Figure 2B (right panel) shows the DMW of the BX1.5 strain recovered from 50 mL of culture medium, which grew to 1.83 g-DMW/L-culture/3 days (0.61 g-DMW/L-culture/day) in a pH 4 medium under 2.0% CO_2_ conditions (Table 1), almost the same as when grown on BG11 adjusted to pH 8 and 11. This finding indicates that BX1.5 strains can grow at pH 4–11 in the BG11 medium. Comparing the DMW on day 3, a 7-times higher biomass production was observed in the 2.0% CO_2_ supply than in the 0.04% CO_2_ under all pH conditions. Thus, acidic and alkaline BG11 liquid media were effective for the indoor culture of the BX1.5 strain, and the medium supplied with 2.0% CO_2_-gas in a “filling system” was suitable for biomass production. As described above, this is the first study on the relationship between the initial pH setting of the medium, DCDC, and biomass production over time in indoor culture for *Parachlorella* sp. In addition, the biomass production of this strain in strongly acidic (pH 4) and strongly alkaline (pH 11) media was maximal compared to that of other green algae.

### 3.2 Outdoor cultivation conditions

To prevent bacterial contamination in outdoor culture, the pH setting of the culture medium was limited to two conditions, acidic and alkaline, which yielded promising results in indoor culture. We then investigated different outdoor culture conditions (Table 1), using only liquid culture or BX1.5 strains in a combination of acidic (pH 3) and alkaline (pH 11) medium, and 0.04% and 2.0% CO_2_ supplied to the BG11 medium in a blowing system (Figure 1B). First, the “cell-free” outdoor medium was filled with 100 L of BG11 culture medium per a VPR and incubated while blowing 60 L of air or CO_2_ gas/min from the bottom. The initial pH of the medium was 3 or 11, and the amount of dissolved carbon dioxide in the medium and the changes in pH were measured continuously. Samples were collected each time by collecting 1 L of the culture medium from the pipe reactor. When cultured without cells, the pH 11 medium decreased to approximately pH 7, as in the indoor culture, whereas the pH 3 medium remained nearly unchanged (Figure 3A). DCDC was the highest in the medium prepared at pH 11 (0.04%, 168.6; 2.0%, 213.0 mg/L-culture/7 days) and was 6.8 times higher than in the medium prepared at pH 4 (0.04%, 28.3; 2.0%, 213 mg/L-culture/7 days).

BX1.5 strains were cultured outdoors with BX1.5 cells to measure changes in medium pH, DCDC, and biomass (Figure 3B). Alkaline (pH 11) and strongly acidic (pH 3) BG11 media showed stable growth under outdoor culture conditions with a 2.0% CO_2_ gas supply, the same as in the indoor culture system. In particular, the biomass reached a maximum of 0.83 g-DMW/L-culture/7 days (0.12 g-DMW/L-culture/day) in pH 3 BG11 liquid medium (Table 1), equivalent to twice the conventional yield of the BX1.5 outdoor culture. In contrast, the alkaline BG11 liquid medium also showed good growth in both cases, with 0.42 g/L-culture on day 7 and 1.0 g-DMW/L-culture on day 14.

This is the first study to show a green alga, *Parachlorella*, thriving in outdoor culture at both alkaline (pH 11) and strongly acidic (pH 3) conditions. The reason underlying the higher biomass in the acidic culture of BX1.5, despite the higher DCDC value on the alkaline medium, is currently unknown and warrants further investigation. Although hydrochloric acid was used to adjust the acidic medium in this study, it is possible that the H^+^ or Cl^+^ ions ionised in the BG11 liquid medium effectively acted on photosynthesis and respiration reactions, resulting in increased biomass production. *Chlorella vulgaris* can grow in an initial medium pH range of 4–9, with maximum specific growth rates observed under pH 5 but little growth under pH 3 conditions (Lam and Lee, 2012). Furthermore, although *C. vulgaris* shows maximum growth rates at pH 10, most algae are pH-sensitive, and few strains have been reported to be able to grow over a wide pH range like *C. vulgaris* (Khan et al., 2018). Therefore, the ability of BX1.5 to grow in a pH of 3–11 and the high potential to grow in medium pH conditions can reduce (or suppress) bacterial growth, which is often a problem in large-scale cultures. Of note, microscopic observation showed almost no bacterial contamination in the outdoor culture. Despite minimal contamination observed under microscopic observations (Figure 4A), there was no change in the colour or odour of the BX1.5 medium (Figure 1B). These reults indicated that BX1.5 can be grown indoors and outdoors, producing useful materials for algal biorefineries. For these reasons, the sugars (carbohydrates) and lipids produced from BX1.5 grown in outdoor culture were characterised as described in the following sections.

### 3.3 bxEPS production indoors and outdoors

Although the BX1.5 strain grew stably under strongly acidic or alkaline conditions, microalgae generally vary greatly in their productivity of useful substances depending on the culture conditions (Singh et al., 2014; Singh et al., 2016; Khan et al., 2018). From our previous reports, BX1.5 strains grown indoors in BG11-N (BG11 without nitrogen source) and BG11-NP (BG11 without nitrogen and phosphorus sources) liquid media with 2.0% CO_2_-filled supply produced high levels of bxEPS as useful extracellular polysaccharides (Sasaki et al., 2020; Sasaki et al., 2021). Therefore, in this study, bxEPS productivity was examined under the outdoor cultivation conditions of BG11 liquid medium at pH 3 or 11 and CO_2_ supply (0.04 or 2.0%), as follows. The BX1.5 cells grown outdoors were harvested and stained with India ink to detect bxEPS (Figure 4A). From the observed cells, 20–25 cells were randomly selected, and the cell diameter (Φ*ic*) and diameter of the outer unstained part (bxEPS) of the cell (Φ*ec*) were measured (Sasaki et al., 2020; Sasaki et al., 2021). The bxEPS production rate was determined as the ratio of Φ*ic* to Φ*ec*. BX1.5 strains cultured outdoors for 14 days in BG11 liquid medium had an average cell diameter of 5–6 µm and an EPS diameter of 8–9.5 µm in the area not stained by India ink under all conditions. Whereas the total bxEPS accumulation was higher in the 2.0% CO_2_ -supplied biomass than that in the 0.04% CO_2_, the bxEPS production expressed as a ratio in Φ*ic* to Φ*ec* per cell was almost the same at pH 3 and 11 (Figure 4B). Therefore, medium pH may not affect extracellular polysaccharide productivity when BG11 medium is used in outdoor cultures. Of note, for the first time, we could confirm a significant accumulation of bxEPS under outdoor conditions in *Parachlorella* sp. strains.

The amount of sugar accumulated from the cells associated with increased biomass production was examined. The biomass (dry cell matters, DCM: cells + bxEPS) was 3.6 or 6.2 times higher (pH 3 or 11) in the 2.0% condition than in the 0.04% condition (Figure 3B), and the sugar accumulation was 3.0 or 3.2 times higher (Figure 5A). The positive correlation between the amounts of accumulated sugars and biomass suggests that CO_2_ fixation through photosynthesis is more efficient with a high supply of CO_2_ concentrations, resulting in increased sugar and biomass accumulation. This information may also be necessary for producing functional extracellular polysaccharides (Mao et al., 2008; Lee et al., 2010; Karnjanapratum and You, 2011; Ngatu et al., 2012; Choi et al., 2015). Overall, these results suggest that efficient biomass accumulation leads to significant sugar accumulation through CO_2_ gas fixation. The monosaccharide composition of the DCM, which consisted of fractions containing cells and extracellular polysaccharides, was dominated by rhamnose and xylose, while glucose and fructose were mainly found in the rest of the DCM (Figure 5B). In addition, an accumulation of galactose was observed in the case of indoor culture. These results are consistent with our previous analysis of the monosaccharide composition of indoor-derived bxEPS (Sasaki et al., 2021). It is remarkable that the strain BX1.5 could produce valuable sugars when cultured both indoors and outdoors. The sugar production per unit medium in outdoor culture ranged from approximately 1–4.5 mg/L-culture (Figure 5A), although the values tended to be higher at lower values of sugar mg/g-DMW under respective culture conditions (Figures 5A,B). This may be due to the cell density per unit medium being lower in outdoor culture than in indoor culture; of note, the amount of sugar produced was relatively high. Nonetheless, for the first time, we showed that outdoor culture under a 2% CO_2_ gas supply is beneficial in terms of sugar productivity per unit culture medium initially set to strong acid and strong alkali conditions up to 14 days of culture (Figure 5A).

### 3.4 Lipid production in indoor and outdoor systems

Studies have been conducted on the production of lipids, such as biofuels and edible oils, using photosynthetic microorganisms. Lipid production is closely linked to intracellular sugar production and is an important part of carbon metabolism (Chisti, 2007; Fan et al., 2011; Li et al., 2013; Mizuno et al., 2013; Shiratake et al., 2013; Xue et al., 2018). However, there have been few studies on lipid production and lipid composition in green algae, especially at different pH and carbon dioxide concentrations in outdoor conditions. We then analysed the amount and composition of lipids accumulated in BX1.5, derived from indoor and outdoor cultures (Figures 2, 3), incubated at each pH to evaluate the effect of pH conditions and dissolved CO_2_ concentration on lipid productivity. The TLC analysis of intracellular lipid components results are shown in Figure 6A. Bands with R*f* values of approximately 0.85–0.9 are considered derived from wax esters (WEs), 0.5 from triacylglycerides (TAGs), and 0.2–0.25 from FFA (Fan et al., 2011; Shiratake et al., 2013; Sasaki et al., 2020; Sasaki et al., 2021). Comparing the composition of accumulated lipids in each sample revealed differences in lipid composition depending on the culture conditions. Notably, in the samples derived from the outdoor culture, a high FFA and WE percentage was observed in the pH 3 condition, and week intensity bands were in the position of TAG (Figure 6B), whereas at pH 11, FAA was the main component, with thin or almost no bands observed in the WE and TAG areas. In contrast, in the indoor culture, although no significant difference was observed between the pH conditions, the bands of TAG were thin, and few triglycerides were produced until day 3 of culture, whereas a significant accumulation of triglycerides was observed in the sample cultured for 6 days. The BX1.5 strain can significantly accumulate triglycerides as its main component on days 4–6 of indoor culture (Sasaki et al., 2020; Sasaki et al., 2021). This finding of these pH-independent changes in lipid composition between outdoor and indoor cultures (FFA > WA >> TAG outdoors at day 14 *versus* TAG > WA >> FAA indoors at day 6) is a first for a photosynthetic microorganism and is a relevant reference for the regulation of lipid production. Possible reasons for changes in lipid composition include light quality (sunlight or artificial light) and CO_2_ gas supply (blown in or filled in).

GC-FID then analysed the composition of the total lipids accumulated in the BX1.5 strain. The results of FAMEs accumulation per litre of culture medium (mg/L-culture) and per dry mass weight (mg/g-DMW) are shown in Figure 7. The significant accumulation of FAMEs as C16:0, C18:2 (ω6), and C18:3 (ω3) was observed in the samples derived from outdoor or indoor conditions under pH 3 and 11 conditions. However, the lipid composition of the BX1.5 strains cultivated outdoors at pH 3 had higher percentages of C16:0, C16:1, and C18:0, whereas those cultivated at pH 11 had higher percentages of C16:3 and C18:3. This finding is consistent with a previous report showing that pH affects lipid composition (Breuer et al., 2013).

On day 3 of indoor culture, little effect of pH conditions on lipid composition was observed, but some differences in lipid productivity per culture medium and dry weight for each pH condition were noted. First, lipid production per culture medium was 130.2 mg/L-culture when cultured in the pH 4 BG11 medium, approximately 1.2 times higher than 108.3 mg/L-culture at pH 8 (Figure 7). Lipid production per DMW similarly increased by approximately 1.08-fold. Still, no difference in biomass per pH condition in the BG11 medium was observed (Figure 2), and cell size did not change (data not shown), suggesting that the lipids in the cells or the extraction efficiency increased while the cell size remained the same. The higher biomass at pH 4 than that at pH 8 (Table 1) suggests that lipid productivity per culture medium was maintained owing to increased cell number, even though lipid accumulation per cell decreased. However, at pH 11, which showed significant differences only per DMW, no significant differences in biomass or cell size compared to pH 8 were observed.

Effective growth and lipid accumulation in *Chlorella vulgaris* was observed at medium pH 7.5 in the range from pH 5 to pH 8, growing “indoor” and “heterotrophically” under a sulfur-restricted medium (Sakarika and Kornaros, 2016; Sakarika and Kornaros, 2017). This study suggests the BX1.5 strain as a promising strain for biorefinery because it can be efficiently cultured outdoors to produce useful sugars and lipids at strongly acidic or alkaline initial pH settings. Generally, inducing sugars and lipids due to nutrient depletion in the medium improves the production effect per unit biomass. However, this often reduces the overall production of valuable products because of the biomass production per unit medium volume under cultivation conditions. Therefore, setting the initial pH of the medium and optimising the dissolved carbon dioxide under “autotrophic” culture conditions in a simple environment, as in the present study, may contribute considerably to biorefineries using microalgae. In the future, it is desirable to strengthen comprehensive process engineering, including efficient recovery and extraction of biomass and valuable products from large-scale indoor and outdoor cultures, as well as processing and manufacturing processes.

## 4 Conclusion

The green alga, *Parachlorella* sp. BX1.5 can effectively grow and produce valuable sugars, such as rhamnose, xylose, and glucose, as well as lipids, such as C16:0, C18:2 (ω6), and C18:3 (ω3), under indoor and outdoor culture conditions when supplied with 2.0% CO_2_ gas in a pH of 3–11. This is the first report of the successful large-scale outdoor cultivation of the green alga *Parachlorella*. The significant production of extracellular polysaccharides (bxEPS) was maintained even when the initial pH was set to either strongly acidic or strongly alkaline conditions, and at the same time, valuable lipids accumulated intracellularly. This remarkable feature of the BX1.5 strain can promise new biorefinery possibilities under outdoor or indoor large-scale culture conditions supplied at high CO_2_ concentrations.

## Data Availability

The original contributions presented in the study are included in the article/Supplementary Material, further inquiries can be directed to the corresponding author.
