# Supplementary material for: Efficient pH and dissolved CO2 conditions for indoor and outdoor cultures of green alga Parachlorella
Source: Front Bioeng Biotechnol. 2023 Sep 11;11:1233944. doi: 10.3389/fbioe.2023.1233944 (PMC10520278; doi:10.3389/fbioe.2023.1233944)
Supplement: Supplementary file 1 [file Table1.docx]

Supplementary Material

Efficient pH and dissolved CO_2_ conditions for indoor and outdoor cultures of green alga *Parachlorella*

**Akari Takagi,^1^ Misato Nagao,^1^ Yuya Uejima,^1^ Daisaku Sasaki,^2^ Munehiko Asayama^1,3*^**

^1^College of Agriculture, Ibaraki University, 3-21-1 Ami, Ibaraki 300-0393, Japan

^2^BioX Chemical Industries Co. Ltd., 2-20-11 Inokuchidai, Nishi-ku, Hiroshima 733-0844, Japan

^3^United Graduate School of Agricultural Science, Tokyo University of Agriculture and Technology, 3-5-8 Fuchu, Tokyo 183-8509, Japan

*** Correspondence:**Munehiko Asayama
E-mail address: [munehiko.asayama.777@vc.ibaraki.ac.jp](mailto:munehiko.asayama.777@vc.ibaraki.ac.jp)

# Supplementary Data

**Table S1 Culture conditions and biomass obtained from the culture**
